# Polygenic modeling of genetic effects on both phenotypic mean and variance: distributional regression for BMI, blood and urine biomarkers in the UK Biobank

**DOI:** 10.3389/fbinf.2026.1800403

**Published:** 2026-06-11

**Authors:** Kiran Kunwar, Qiong Wu, Hannah Klinkhammer, Christian Staerk, Andreas Mayr, Carlo Maj

**Affiliations:** 1 Center for Human Genetics, Marburg University, Marburg, Germany; 2 Institute for Medical Biometry and Statistics, Marburg University, Marburg, Germany; 3 Institute for Genomic Statistics and Bioinformatics, University of Bonn, University Hospital Bonn, Bonn, Germany; 4 IUF – Leibniz Research Institute for Environmental Medicine, Düsseldorf, Germany; 5 Department of Statistics, TU Dortmund University, Dortmund, Germany

**Keywords:** biomarkers, distributional regression, genetic epidemiology, linkage disequilibrium, polygenic risk score, prediction model, UK Biobank

## Abstract

Polygenic scores (PGS) are commonly used to estimate the cumulative genetic contribution to complex traits by aggregating the effects of multiple genetic variants. In the case of continuous phenotypes, traditional methods have primarily focused on predicting the effect of variants on the phenotypic mean, thereby overlooking potential genetic influences that modulate phenotypic variance. Recent studies suggest that variance quantitative trait loci (vQTLs) provide important insights into the genetic control of trait variability and may serve as candidates for gene–environment interactions that are not captured by mean-based models. Using UK Biobank data, we applied *snpboostlss*, a cyclical gradient-boosting framework for Gaussian location–scale models, to derive sparse polygenic models for both the mean and the variance of quantitative traits simultaneously. We analyzed BMI and 30 blood and urine biomarkers (e.g., cholesterol, glucose, phosphate, urate) where joint genetic, environmental, and lifestyle contributions (e.g., sedentary behavior, diet) are expected. The distributional regression approach efficiently processes large-scale and high-dimensional genotype data by selecting, in each boosting iteration, a batch of variants that exhibit strong correlations with the current residuals. By estimating genetic effects on both the mean and the variance of quantitative traits, *snpboostlss* allows the construction of dual-component polygenic models that offer a more detailed view of the genetic architecture underlying the trait. Our analyses revealed genetic loci that influence the level of the trait alongside markers that predominantly affect trait variability. Notably, some variants contributed to both components, while others were specific to variance, suggesting distinct mechanisms and potential evidence for gene–environment interplay. These findings demonstrate that integrating variance effects into polygenic modeling via distributional regression can improve model interpretability and yield refined predictive insights for complex traits within precision medicine. Also, by including a variance component, our model has the potential to stratify individuals who could particularly benefit from environmental changes and lifestyle interventions.

## 1 Introduction

Polygenic scores (PGS) have become widely used tools in genetic epidemiology to quantify the cumulative genetic contribution to complex traits and diseases (Choi et al., 2020). These scores aggregate effects across many genetic variants identified through genome-wide association studies (GWAS), enabling risk stratification and prediction of phenotypic outcomes at the individual level (Miao et al., 2022). Despite their widespread application, for continuous traits, traditional polygenic models primarily estimate additive genetic effects on the phenotypic mean, leaving a substantial portion of heritability unexplained—commonly referred to as the “missing heritability” problem (Maher, 2008; Manolio et al., 2009; Gibson, 2010). Missing heritability describes the discrepancy between heritability estimates obtained from genome-wide association studies (GWAS) and those measured through family-based studies using twins, siblings and close relatives. The majority of the methods for genetic heritability prediction are based on pure additive effects of common single nucleotide polymorphism (SNP- $h^{2}$ ). GWAS primarily focus on the identification of common genetic variants that are statistically significant for diseases or phenotypic traits but account for a small proportion of the overall heritability (Young, 2019). This gap between observed heritability in family studies and the genetic variance explained by PGS is especially pronounced in complex multifactorial traits and may be due to rare genetic variations, non-linear effects (epistasis), gene–environment (GxE) interactions, and other forms of genetic variation that are not captured by currently applied genotyping and sequencing technologies (copy number variations (CNVs), *etc.*) (Zuk et al., 2012; Young, 2019). Unlike common genetic variants that can provide valuable insights on the population-level variability of a phenotype, the effects of rare variants to a trait variability on a population level are often minimal (Weiner et al., 2023). However, these less frequent variants can have strong effect sizes and thus have a major contribution in individual level disease risk especially as it can be observed for monogenic diseases (Rabbani et al., 2014).

Recent research has identified several variance quantitative trait loci (vQTLs) in the human genome, that are genetic variants associated with differences in trait variability across genotype groups (Marderstein et al., 2021; Hill and Mulder, 2010; Ivarsdottir et al., 2017; Yang et al., 2012). These vQTLs may serve as candidates for underlying gene–environment interactions (GxE), as heterogeneity in trait variance could reflect differential genetic susceptibility to environmental exposures (Marderstein et al., 2021; Miao et al., 2022). Therefore, incorporating genetic effects on phenotypic variance into polygenic models might provide more insights on genetic variants associated with the phenotype via gene-environment modulations (Rönnegård and Valdar, 2011; Young et al., 2018; Wang et al., 2019). The extension of classical mean based PGS models estimating the conditional mean of the trait, to the variance based models, that estimate overall genetic effects on the variability of a trait, can capture potential genetic contributions that are associated with the trait plasticity, gaining additional genetic information that is orthogonal to the classical PGS methods (Miao et al., 2022).

To address this, our work employs distributional regression methods (Rigby and Stasinopoulos, 2005; Stasinopoulos et al., 2024) for polygenic modeling, specifically *snpboostlss* (Wu et al., 2026), which is an extension of mean-based polygenic modeling method *snpboost* (Klinkhammer et al., 2023; 2024). The *snpboostlss* algorithm simultaneously estimates genetic effects on both the mean and variance components of complex traits also by incorporating the mutual influence between the two (Equation 1). By applying this approach to body mass index (BMI) data and 30 serum and urine biomarkers from the UK Biobank (Bycroft et al., 2018), we derive both mean polygenic scores (mPGS) and variance polygenic scores (vPGS). Further, our approach can identify loci contributing to both the mean and the variance of traits, distinguish shared and variance-specific genetic effects and hence, could potentially serve as a tool to identify individuals with higher trait variability who might particularly benefit from environmental interventions, such as lifestyle modifications. These findings underscore the importance of incorporating a variance component in PGS modeling to enhance predictive accuracy and provide deeper biological insights into the genetic architecture of complex traits.

## 2 Methods

### 2.1 UK biobank genotypic and phenotypic data processing and analyses

The analyses were conducted using genotype and phenotype data from the UK Biobank, comprising approximately 350,000 unrelated individuals of European ancestry genotyped for over 500,000 genetic variants. Genetic variants were quality-controlled, retaining those with a minor allele frequency (MAF >0.1% ), Hardy–Weinberg equilibrium $\left(p>10^{-6}\right)$ , and low genotype missingness (<10%) . Genome Reference Consortium Human build 37 (GRCh37) was used as the reference genome for all analyses in this study.

In order to investigate the genetic basis of biomarkers on large scale, BMI and 30 available blood and urine biomarkers (Fry et al., 2019; UK Biobank, 2019) from the UK Biobank were chosen as target traits, as these traits are jointly influenced by both genetic and environmental factors. Target phenotype files were prepared by using the corresponding field ID for each trait (Table 1) from the UK Biobank database. Our aim was to apply *snpboostlss* for detection of genetic variants that are associated with the mean and the variance of the phenotypes (mPGS and vPGS variants) and to explore potential gene-environment interactions. We restricted our analyses to unrelated individuals from the UK Biobank (UKBB resource 668) with self-reported White British ancestry and inferred European ancestry (UKBB fields 21000 and 22006). For each trait, individuals with discordant genetic sex and self-reported sex (UKBB fields 22001 and 31) were removed. All participants with available baseline data were retained for the analyses. The resulting individuals were randomly stratified into training, validation, and test sets with a ratio of 2:1:1 and *snpboostlss* was applied on the training and validation sets with the default parameter settings as described in Section 2.2 and Wu et al. (2026).

**TABLE 1:** Prediction performance across BMI and 30 blood and urine biomarkers.

fid	Phenotype_name	Abbr	$R_{\text{test}}^{2}$	$R_{\text{mPGS}-\text{vPGS}}$	$n_{\text{test}}$	$R_{\text{cov}}^{2}$	$R_{\text{full}}^{2}$	Delta	F stat	p_adj
21001	BMI	BMI	4.84e-2	3.99e-1	88,080	9.02e-3	5.06e-2	4.16e-2	1.43	<3.28e-16
30620	Alanine aminotransferase	ALT	1.00e-2	7.32e-1	83,753	6.35e-2	7.37e-2	1.02e-2	2.00	<3.28e-16
30600	Albumin	ALB	4.35e-2	5.98e-4	76,746	3.63e-2	7.67e-2	4.04e-2	0.96	9.07e-2
30610	Alkaline phosphatase	ALP	9.83e-2	1.62e-2	83,802	2.99e-2	1.28e-1	9.77e-2	1.17	2.17e-12
30630	Apolipoprotein A	APOA	9.91e-2	4.61e-1	76,468	1.56e-1	2.56e-1	9.96e-2	1.31	<3.28e-16
30640	Apolipoprotein B	APOB	1.32e-1	6.57e-1	83,427	3.76e-3	1.35e-1	1.32e-1	1.40	<3.28e-16
30650	Aspartate aminotransferase	AST	1.64e-2	2.21e-1	83,864	3.10e-2	4.76e-2	1.66e-2	5.10	<3.28e-16
30710	C-reactive protein	CRP	1.79e-2	5.58e-1	83,828	5.34e-3	2.30e-2	1.77e-2	1.59	<3.28e-16
30680	Calcium	CA	4.38e-2	9.10e-3	76,499	9.59e-3	5.21e-2	4.25e-2	0.99	7.86e-1
30690	Cholesterol	CHOL	8.17e-2	6.29e-1	84,150	3.42e-2	1.14e-1	7.99e-2	1.50	<3.28e-16
30510	Creatinine in urine	UCR	1.07e-3	1.19e-1	85,329	1.08e-1	1.05e-1	−2.91e-3	1.07	2.23e-3
30660	Direct bilirubin	BILD	1.94e-1	9.29e-1	71,404	4.74e-2	2.51e-1	2.03e-1	5.38	<3.28e-16
30730	Gamma glutamyltransferase	GGT	1.71e-2	5.71e-1	83,390	3.52e-2	5.21e-2	1.68e-2	2.18	<3.28e-16
30740	Glucose	GLU	9.37e-3	2.90e-1	76,617	1.52e-2	2.45e-2	9.30e-3	1.81	<3.28e-16
30750	HbA1c	HBA1C	3.55e-2	2.84e-1	83,569	4.17e-2	7.73e-2	3.56e-2	1.94	<3.28e-16
30760	HDL cholesterol	HDL	1.19e-1	6.73e-1	76,611	1.74e-1	2.93e-1	1.18e-1	1.81	<3.28e-16
30770	IGF-1	IGF1	8.88e-2	1.55e-1	83,696	7.12e-2	1.58e-1	8.63e-2	1.06	1.37e-2
30780	LDL cholesterol	LDLD	9.25e-2	6.72e-1	83,696	8.66e-3	1.00e-1	9.14e-2	1.59	<3.28e-16
30790	Lipoprotein A	LPA	5.27e-1	7.36e-1	66,857	8.85e-4	5.30e-1	5.29e-1	8.59	<3.28e-16
30500	Microalbumin in urine	URMA	5.42e-6	2.13e-1	26,337	2.14e-3	−1.25e-4	−2.26e-3	2.97	<3.28e-16
30810	Phosphate	PHOS	4.09e-2	−4.26e-2	76,474	5.92e-2	9.75e-2	3.84e-2	1.02	5.32e-1
30520	Potassium in urine	URK	2.15e-4	5.31e-2	85,401	2.09e-2	1.86e-2	−2.26e-3	1.02	3.18e-1
30830	SHBG	SHBG	6.97e-2	6.77e-1	76,386	1.65e-1	2.35e-1	6.97e-2	1.58	<3.28e-16
30530	Sodium in urine	URNA	2.79e-3	1.62e-1	84,677	7.22e-2	6.67e-2	−5.49e-3	1.05	3.88e-2
30850	Testosterone	TES	5.05e-3	6.12e-1	76,526	8.07e-1	8.09e-1	2.07e-3	1.72	<3.28e-16
30840	Total bilirubin	TBIL	3.02e-1	9.64e-1	83,588	6.09e-2	3.62e-1	3.01e-1	9.16	<3.28e-16
30860	Total protein	TP	5.91e-2	7.00e-3	76,421	4.62e-3	6.07e-2	5.61e-2	1.04	7.59e-2
30870	Triglycerides	TRIG	8.09e-2	8.71e-1	83,731	4.73e-2	1.28e-1	8.03e-2	3.89	<3.28e-16
30880	Urate	UA	7.51e-2	6.64e-2	83,731	2.79e-1	3.52e-1	7.32e-2	1.23	<3.28e-16
30670	Urea	BUN	2.29e-2	1.29e-2	83,885	7.75e-2	9.93e-2	2.17e-2	0.99	9.62e-1
30890	Vitamin D	VITD	4.39e-2	7.76e-1	80,158	9.61e-3	5.08e-2	4.12e-2	2.14	<3.28e-16

$R_{\text{test}}^{2}$ : squared Pearson correlation between predicted mPGS and true phenotype, $R_{\text{mPGS}-\text{vPGS}}$ : Pearson correlation between predicted scores from mPGS and vPGS model on the test set, $n_{\text{test}}$ : number of individuals in the held-out test set, $R_{\text{cov}}^{2}$ : predictive performance of the model with covariates (age, sex and PCs) alone, $R_{\text{full}}^{2}$ : predictive performance of the full model (including covariates and mPGS), delta: incremental predictive performance of the full model against the covariate-only model, F stat: ratio of variances between high vPGS and low vPGS groups, p_adj: Benjamini–Hochberg FDR adjusted two-sided p-values from F statistics.

### 2.2 Boosting distributional regression for PGS estimation

We modeled genetic effects on the phenotypes using Gaussian location-scale models. Let $y={\left(y_{1},\ldots ,y_{n}\right)}^{⊤}$ denote the vector of phenotype measurements from n individuals, and let $X\in R^{n\times p}$ represent the genotype dosage matrix, where p denotes the number of genetic variants. We consider the following distributional regression model (Stasinopoulos et al., 2024):

$$y_{i}∼N\left(\mu_{i},\sigma_{i}^{2}\right),\;\mu_{i}=\beta_{0}+\overset{p}{\underset{j=1}{\sum }}\beta_{j}x_{i,j},\;\log\left(\sigma_{i}\right)=\gamma_{0}+\overset{p}{\underset{j=1}{\sum }}\gamma_{j}x_{i,j}.$$ (1)

The objective is to simultaneously estimate coefficients $\beta ={\left(\beta_{0},\ldots ,\beta_{p}\right)}^{⊤}$ and $\gamma ={\left(\gamma_{0},\ldots ,\gamma_{p}\right)}^{⊤}$ by minimizing the loss function defined as the negative log-likelihood and model genetic effects on both location (mean) and scale (variance) components of the trait. For high dimensional genetic data, component-wise gradient boosting (Bühlmann and Hothorn, 2007) can select variables and estimate coefficients simultaneously by specifying a loss function $\rho \left(y,\hat{y}\right)$ and the so-called base learners $h_{j},\;j=1,\ldots ,p$ , that are iteratively fitted to the response (Klinkhammer et al., 2023; Klinkhammer et al., 2024). In each iteration, once the best fitting base learner is selected, the algorithm updates the corresponding predictor. Such updating process stops when a maximum number of boosting iterations (default 1,000) is reached.

Due to the presence of linkage disequilibrium (LD) blocks, genetic signals are often shared among correlated variants (i.e., variants in LD). This redundancy allows the underlying association signal to be effectively captured by a subset of representative variants, enabling efficient polygenic modeling in high-dimensional settings. Consequently, sparse regression methods with built-in feature selection can be employed, resulting in models where most coefficients in β and γ are set to zero. This modeling strategy reflects the expected genetic architecture, in which a limited number of causal variants generate association signals within a given locus that propagate to nearby variants through LD, allowing redundant predictors to be excluded without substantial loss of information.

Due to the substantial dimensionality ( n≈350,000 , p≈500,000 ), direct fitting of standard Gaussian location-scale models is computationally infeasible. To overcome this, we applied the *snpboostlss* algorithm (Wu et al., 2026), which adopts batch-wise gradient boosting on large genetic data for distributional regression. After rigorous quality control followed by random stratification into training, validation, and test sets (2:1:1), the training set was used to fit the model, the validation set to define the stopping iterations and the test set to measure the overall predictive performance of the model. The algorithm consists of an outer loop and an inner loop. The outer loop corresponds to the batch-building procedure, where we extract the $p_{\text{batch}}$ variants ( $p_{\text{batch}}\ll p$ , default $p_{\text{batch}}=1,000$ ) with the highest correlation to the current negative gradient vectors on modeling *µ* and σ to form separate batches for *µ* and σ respectively. Then, the algorithm enters the inner loop to sequentially update coefficients for *µ* and σ via cyclical boosting with these constructed variant batches for a maximum number of iterations (default 1,000 iterations). Early stopping of boosting for either location or scale parameter is allowed if there exists a variant outside the batch showing a higher correlation with the negative gradient vectors compared to all variants inside the batch. If boosting is stopped early for one parameter, the other will keep being updated until the stopping criteria for the inner loop are met. The inner loop gets terminated when either both parameters have been early stopped or the maximum number of boosting iterations is reached. Once the inner loop has been completed, we return to the outer loop to rebuild batches and repeat the process. In total, we fit a predefined maximum number of batches (default 20,000) or stop the algorithm early if the fitted model does not show improvements on the validation set. Specifically, we simultaneously monitor the predictive performance of our model on the independent validation set, using the loss as the evaluation criterion. The outer loop will be early stopped if the loss on the validation set has not decreased for two consecutive batches (representing the default value). A detailed description of the algorithm and simulation results is described in Wu et al. (2026).

### 2.3 Analysis of mPGS and vPGS

The mPGS and the vPGS models were trained on the individuals from the UK Biobank using *snpboostlss* with default values as described in Wu et al. (2026). While the mPGS models predict the mean of the phenotype dependent on the genetic information, vPGS models stratify individuals according to their genetically inferred propensity for phenotypic variability, as the model is trained using variants associated with variance effects rather than mean effects. To assess the overall contribution of predicted scores on trait mean and variability, we first characterized the phenotypic distribution across the estimated mPGS and vPGS deciles for all traits. Then, we quantified the relationship between predicted scores from the mPGS and vPGS models for each trait based on the correlation (Pearson) between these two models on the independent test set. Here, the goal was to further investigate to which extent the genetic architecture of a trait can be explained by genetic predisposition towards the mean of the phenotype or influenced by genetic component associated with the phenotypic variability (i.e., to what extent the genetic influences for the trait’s variability are related to the genetic influences for the trait’s mean). Next, we evaluated the prediction performance of the mPGS model. To this end, we report i) the genotype-only model, where we used the plink2 (Chang et al., 2015) computed polygenic scores (--score command) for each individual from the held-out test set and calculated the squared correlation $\left(R^{2}\right)$ between predicted and observed phenotypes (Staerk et al., 2024), ii) the covariate-only model, where we fit a regression model $\left(Y∼\text{age}+\text{sex}+{\text{PC}}_{1-10}\right)$ to compute the variance explained by non-genetic covariates alone, and iii) the full model to compute the variance explained by both mPGS and covariates $\left(Y∼\text{mPGS}+\text{age}+\text{sex}+{\text{PC}}_{1-10}\right)$ . Also, we derived the incremental predictive performance (delta) by computing the difference in $R^{2}$ between the full and the covariates-only models. Further, to investigate the phenotypic variability on vPGS extremes, we stratified the individuals into high/low vPGS groups by taking the top 10 and the bottom 10 percentile of the predicted vPGS from the held-out test set respectively. Individuals on the top vPGS groups are expected to exhibit more genetic variability for a trait, whereas those in the bottom vPGS groups are expected to show lower variability. Then, to understand the differences in phenotypic variability between top vPGS and bottom vPGS groups, we first calculated the sample variance using an unbiased estimator with n−1 degrees of freedom and computed F-test statistics (Box, 1953; Lomax, 2007) by comparing the ratio of variances between these extreme vPGS groups. Next, we calculated two-sided p-values from the F-distribution defined as:

$$p=2\times \min\left[P\;\left(F_{{\text{df}}_{1},{df}_{2}}\leq F_{\text{obs}}\right),\;1-P\;\left(F_{{\text{df}}_{1},{df}_{2}}\leq F_{\text{obs}}\right)\right]$$

where

$${df}_{1}=n_{\text{top}}-1,\;{df}_{2}=n_{\text{bottom}}-1,\;F_{\text{obs}}=\frac{{variance}_{\text{top}}}{{variance}_{\text{bottom}}}.$$

P-values from two-sided tests were adjusted for multiple testing using the Benjamini–Hochberg procedure controlling the false discovery rate (FDR).

### 2.4 LD computation

For all selected variants from either the mPGS or the vPGS models (“query variants”), we computed the pairwise linkage disequilibrium by using LDlink API (Machiela and Chanock, 2015). To investigate the pattern of LD between variants, we used the module LDmatrix, that includes FORGEdb scores (used to evaluate functional importance of genetic variants) (Breeze et al., 2024) for the functional annotation of regulatory variants. Briefly, LDmatrix takes the user supplied list of SNPs as input, matches them with dbSNP rsids (Phan et al., 2025; Sherry et al., 2001) and computes the pairwise LD between all input pair summarized by the squared correlation coefficient between two variants $\left(R^{2}\right)$ and the normalized LD coefficient $\left(D^{\prime }\right)$ . As a result, a single symmetric LD matrix containing the pairwise LD for all query variants is returned. Publicly available reference haplotypes from the 1000 Genome Project Phase 3 (The 1000 Genomes Project Consortium, 2012) with genome build hg19 (GRCh37) for European population was used as a reference population, as this study is based on the White British individuals from European descent. For each trait and chromosome, we created a list of query variants per chromosome by combining the complete set of variants from the mPGS and vPGS models that are selected for that chromosome and computed chromosome-wise LD between these variants to derive an $N_{c}\times N_{c}$ matrix per chromosome, where $N_{c}$ represents the total number of distinct selected variants for chromosome c , and each entry in the matrix corresponds to the correlation value ( $R^{2}$ or $D^{\prime }$ ) for one variant-variant pair. The dimension of the constructed squared matrix is equal to the number of query variants. For our analysis, we used $R^{2}$ metrics for LD calculation and a mPGS variant is tagged as a variant in linkage with a vPGS variant, if $R^{2}\geq 0.5$ for these variants and *vice versa*. The resulting chromosome-wise LD-matrices were used to characterize the correlation structure among selected variants and to define the LD status of variants representing mPGS and vPGS models. Finally, the variants with $R^{2}\geq 0.5$ were tagged as “variants_in_LD”.

### 2.5 Analysis of overlaps between variants, LD-linked variants, and genes

Variants that were selected by our mPGS and vPGS models were matched to genes according to genomic coordinates. ANNOVAR (2019 October 24 release) was used to perform gene-based annotation and to functionally annotate the selected variants. Variants located in coding and intronic regions were assigned to the corresponding gene. For variants residing in the intergenic region, the two closest flanking genes corresponding to the variant location were taken into account for the annotation. We evaluated the overlap between mPGS and vPGS at multiple biological resolutions: individual variants, annotated genes, and LD blocks. To perform the variant-based overlap analysis, we examined, for all traits, the model-selected variants and categorized them into three different variant sets based on their effect size: i) mPGS variants–variants with coef. *µ* ≠ 0; ii) vPGS variants–variants with coef. σ≠ 0; and iii) shared variants–variants with coef. *µ* ≠ 0 *and* coef. σ≠ 0. Then, we conducted the overlap analysis between the mPGS, vPGS and shared variants on the level of LD structure by taking the pairwise linkage disequilibrium statistics ( $R^{2}$ ) between each variant pair that is selected by mPGS and vPGS models into account. The variant overlap analysis is followed by the overlap analysis at gene-based level. Here, we stratified the gene sets specific to the mPGS and vPGS models along with the annotated gene loci that are shared between these models, and investigated their association with the corresponding traits.

### 2.6 Gene enrichment analysis

Next, for each trait, we derived annotated gene sets that are specific to our mPGS and vPGS models and performed gene enrichment analysis on the selected mPGS and vPGS gene sets using Enrichr (Kuleshov et al., 2016) (2023 June 8 release). Briefly, Enrichr implements Fisher’s exact test on many random gene sets to compute a mean rank and standard deviation from the expected rank for each term in each gene-set library. The resulting p-values are adjusted for multiple testing using Benjamini–Hochberg procedure for FDR correction. Terms are ranked based on z-score that is derived by assessing the deviation from the expected rank. To achieve this, we first prepared gene sets for each trait that were specific to the mPGS and the vPGS models with common annotated genes present in both sets. The aim was to investigate whether the annotated genes linked to variants that are predicted by our sparse mPGS and vPGS framework are significantly enriched for terms related to predefined biological pathways. Specifically, for genes that were linked to variants identified by our vPGS model, we investigated the enrichment of these genes on external exposures such as response to environmental factors potentially hinting to a gene-environment interplay. The enrichment results of the mPGS and vPGS genes were examined on terms reported in the GWAS catalog 2025 (Cerezo et al., 2025).

## 3 Results

### 3.1 Analysis of the mPGS and vPGS models

We investigated the relationship between predicted mPGS and vPGS for the individuals passing quality control and observed a wide variation of correlation between mPGS and vPGS across all traits that ranged from near zero ( $5.9\times 10^{-4}$ for ALB) to near one (0.96 for TBIL) (Supplementary Table S1). Here, Pearson correlation coefficient (R) was used as the correlation metric. The results clearly indicate the trait-specific heterogeneity in the correlation between genetic influences that are associated with the mean and the variability of a trait. Those blood and urine biomarkers exhibiting near zero correlation between the mPGS and vPGS suggest independent genetic architectures that are driving the mean and the variance of these traits, whereas those having stronger (≥0.7) correlation indicate the shared and colocalized effects of genetic components on traits estimated by mPGS and vPGS models. Likewise, biomarkers showing a weak to moderate correlation (e.g., UCR: 0.11; GLU: 0.29; CHOL: 0.62) are those with shared genetic signals between mPGS and vPGS models and are also likely exhibiting considerable trait heterogeneity (Miao et al., 2022). Since variants can have joint effect on both the mean and the variance of the trait, and also due to the presence of linkage disequilibrium between the mean and the variance associated variants, a complete orthogonality between mPGS and vPGS models is not expected.

By comparing the ratio of phenotypic variance between the top and bottom vPGS quantiles, the observed F-test statistics were greater than 1 for 28 out of 31 traits, indicating that vPGS effectively stratified individuals by trait variability (Table 1). Notably, for the remaining three traits, ALB, CA and BUN, the F-test statistics value comprised 0.96, 0.99 and 0.99 respectively, meaning that the individuals grouped under lower vPGS groups exhibited higher variability compared to those from the higher vPGS groups. However, there were no statistically significant differences in the variance between these two groups (adjusted p-value comprised 0.09, 0.78 and 0.96 respectively, (Table 1)). A higher phenotypic variability among subjects representing the top vPGS groups hints that the elevated phenotypic heterogeneity is contributed by the genetic predisposition of individuals that is associated with the variance of the phenotype.

### 3.2 Prediction performance of the PGS models

**FIGURE 1:**
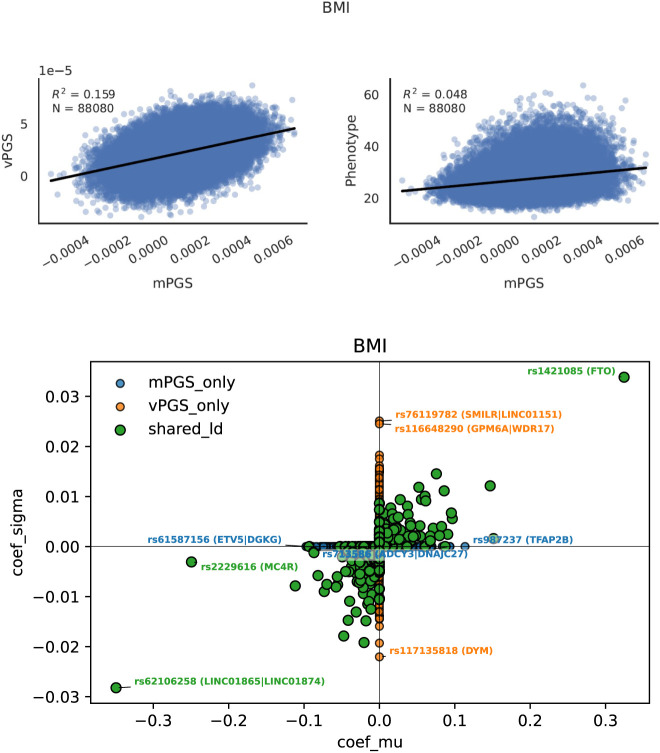
Correlation between the mean-based mPGS and the variance-based vPGS models **(upper panel left)** and the correlation between the predicted mPGS values and true phenotype **(upper panel right)** for BMI. *snpboostlss* predicted genes based on the effect size of associated variants **(lower panel)**. Genes are categorized into three different groups: mPGS genes that are associated only with the mean BMI (blue dots), vPGS genes that are associated only with the variance of BMI (orange dots) and shared genes common between mPGS and vPGS that are associated with both BMI mean and variability (green dots). Variants from only mPGS model (x-axis) that are in linkage with the variants from only vPGS model (y-axis) are also designated as shared LD variants (green dots). Top three genes from each category are annotated with reference to human genome build 19 (GRCh37). For genes associated with multiple variants, the top variant with maximum effect size is chosen for the plot.

We constructed PGS for BMI and 30 blood and urine biomarkers using a cyclical gradient-boosting framework implemented in *snpboostlss*. The prediction accuracy of the mPGS models was measured as the squared Pearson correlation $\left(R^{2}\right)$ between the true phenotypic value and the predicted score for the corresponding phenotype (mPGS) on the held-out test set, which is a commonly used metric in the field of polygenic risk modeling to evaluate the performance of the polygenic models (Lewis and Vassos, 2020; Qian et al., 2020; Wand et al., 2021; Momin et al., 2023). The predictive performance of the constructed mPGS models was quantified based on the genotype features alone. On average across all 31 traits, the constructed mPGS models showed a modest predictive performance, explaining low to moderate trait variability on the independent test set of White British descent from the UK Biobank (mean $R^{2}=0.08$ , median $R^{2}=0.04$ ) (Table 1). However, we also observed a significant heterogeneity on the predictive accuracy of the mPGS models across the analyzed serum and urine biomarkers. For the target phenotype BMI, the $R^{2}$ between the estimated mPGS and true phenotype was 0.048 (Figure 1; Table 1), meaning that the constructed mPGS model can explain up to 4.8% of the BMI variability in the independent test set. After fitting the linear regression model, the predictive performance of the covariates-only model (including age, sex, and the first ten PCs) and the full model (including PGS and aforementioned covariates) for BMI was 0.009 and 0.05 respectively, resulting in an incremental predictive performance of 0.041 (Table 1). Since *snpboostlss* allows the simultaneous modeling of mPGS and vPGS (Equation 1), we then checked the correlation between the predicted scores for mPGS and vPGS models. For BMI, the Pearson correlation between the mPGS and vPGS models ( $R_{\text{mPGS}-\text{vPGS}}$ ) on the held-out test set was 0.39 (Table 1). Consistent with prior results, where the authors implemented penalized regression using Batch Screening Iterative Lasso (BASIL) algorithm (Sinnott-Armstrong et al., 2021; Tanigawa et al., 2022) and used Nagelkerke’s pseudo- $R^{2}$ as a measure of prediction performance, the best predictive accuracy based on $R^{2}$ was obtained for Lipoprotein A explaining up to 52.7% of the trait variability ( $P<1\times 10^{-308}$ , N=66,857 ) (Table 1). Here, the contribution of covariates was very small $\left(R^{2}=0.0008\right)$ (Table 1) indicating that Lipoprotein A is largely driven by genetic components. This is expected, as it has been previously reported that the distribution of Lipoprotein A is largely regulated by genetic variants at the *LPA* gene locus on chromosome 6q27 (Kronenberg and Utermann, 2013). In contrast to Lipoprotein A, where the trait variability is dominated by genetic components, a substantial proportion of phenotypic variability $\left(R^{2}=0.8\right)$ for Testosterone is explained by covariates with a minimal effect of mPGS (delta $R^{2}=0.002$ ) (Table 1), consistent with previous reports (Tanigawa et al., 2022), suggesting a larger influence of non-genetic factors such as sex and age rather than common genetic variants captured by the mPGS model. Similarly, for the inflammatory biomarker C-reactive protein, mPGS explained up to 1.7% of the variance on C-reactive protein levels in the held-out test set ( $P<1\times 10^{-308}$ , N=83,828 ), whereas the prediction accuracy was limited for urine biomarkers such as Microalbumin in urine ( $R^{2}=5.4\times 10^{-6}$ , $P=7.06\times 10^{-1}$ , N=26,337 ), Potassium in urine ( $R^{2}=2.1\times 10^{-4}$ , $P=1.8\times 10^{-5}$ , N=85,401 ), and Creatinine in urine ( $R^{2}=1.07\times 10^{-3}$ , $P=1.08\times 10^{-21}$ , N=85,329 ) (Table 1) likely reflecting their lack of steady-state conditions and lower heritability compared to blood-based traits. The predictive performance $\left(R^{2}\right)$ for the constructed mPGS models and biomarkers, and the cross-correlation between the mPGS and the vPGS models is presented in Table 1 and Supplementary Figure S1 in detail.

### 3.3 Overlap of variants, LD-linked variants, and genes

To describe the downstream analyses including the overlap assessment of *snpboostlss*-selected variants, associated genes and LD-Blocks, we describe overall patterns across blood and urine biomarkers and illustrate the results for BMI in detail. The results for the remaining blood and urine biomarkers are available in Table 2, Supplementary Tables S1, S2 and the Supplementary Material 2. For BMI, *snpboostlss* selected a total of 4,888 variants that are associated with the trait. The model further classified these variants into three categories based on their effect sizes: i) mPGS variants associated with the mean of the trait alone, ii) vPGS variants associated with the variance of the trait alone and iii) shared variants that are common between mPGS and vPGS models and affect both the trait mean and variability. The number of variants predicted for each of the categories comprised 2,081 (42.5%); 2,570 (52.6%) and 237 (4.8%), respectively (Table 2). Notably, the total number of variants selected by *snpboostlss* varies widely across traits ranging from 52 (URMA: Microalbumin in urine) to 4,895 (TP: Total Protein). Overall, for 24/31 traits, the model selected more vPGS variants than mPGS variants (Table 2). For the remaining 7/31 traits (ALP, AST, BILD, BUN, GLU, PHOS, TES), the number of variants selected by the mPGS model was larger in comparison to the number of variants selected by the vPGS model (Table 2). Next, in order to investigate whether the mPGS-selected top variants with larger effect sizes on the trait mean are also identified by the vPGS model as the leading variants with higher influence on trait variability, we examined the top ten variants from each model for all traits based on their effect sizes. To this end, we also took into account the LD structure between the variants from each model. For most of the traits, we observed that the large majority of the top variants predicted by the mPGS model were linked to trait variability, while only a small number of top variants selected by the vPGS model were associated with the trait mean (Supplementary Material 2, Supplementary Table S4). Notably, for traits such as ALB, ALP, BUN, CA and TP, no pairwise LD was detected between the top ranked mPGS and vPGS variants and any other variants that are selected by the model, suggesting that the model prioritized different genetic signals potentially capturing orthogonal genetic information (Supplementary Material 2, Supplementary Table S4). Likewise for BMI, we found that eight leading variants that influence the average BMI were commonly identified by both mPGS and vPGS models, indicating the association of these variants also with BMI variability. Conversely, only four out of top ten vPGS variants were common to mPGS variants. We investigated these four common variants (rs1421085, rs62106258, rs117793826, rs114689744) in greater detail and observed that the former two variants, which had the maximum effect on BMI variance, were also the variants with maximum effect on the BMI mean (Figure 1), whereas the latter two variants were found to have a very low effect on the average BMI. Interestingly, one of the top 4 common variants, rs1421085 (effect allele = C), has a positive effect on both the mean and the variance of BMI, whereas the other variant, rs62106258 (effect allele = C), has a negative effect on both BMI mean and variability respectively (Figure 1). This pattern was quite common on multiple other traits, for example: CRP, HDL, SHBG, TES, TRIG, VITD, with respectively one of the top variant having positive effect while the other having negative effect on the trait (Supplementary Material 2).

**TABLE 2:** Summary of variants, LD-linked variants and genes overlap across BMI and 30 blood and urine biomarkers.

FID	Phenotype name	Abbr	Total variants	mPGS variants	vPGS variants	Shared variants	Variants in LD	Total genes	mPGS genes	vPGS genes	Shared genes	% Shared variants	% variants in LD	% Shared genes
21001	BMI	BMI	4888	2081	2570	237	366	4423	2394	3025	996	4.8%	7.5%	22.5%
30620	Alanine aminotransferase	ALT	67	23	40	4	8	92	27	69	4	6.0%	11.9%	4.3%
30600	Albumin	ALB	3308	1231	2069	8	14	3503	1453	2393	343	0.2%	0.4%	9.8%
30610	Alkaline phosphatase	ALP	330	203	127	0	0	323	151	178	6	0.0%	0.0%	1.9%
30630	Apolipoprotein A	APOA	4245	1586	2561	98	150	4161	1798	2975	612	2.3%	3.5%	14.7%
30640	Apolipoprotein B	APOB	3984	1625	2258	101	186	4001	1921	2639	559	2.5%	4.7%	14.0%
30650	Aspartate aminotransferase	AST	153	105	47	1	2	172	107	66	1	0.7%	1.3%	0.6%
30710	C-reactive protein	CRP	241	66	156	19	28	327	89	262	24	7.9%	11.6%	7.3%
30680	Calcium	CA	1772	675	1094	3	13	2085	764	1421	100	0.2%	0.7%	4.8%
30690	Cholesterol	CHOL	3593	1363	2149	81	150	3646	1593	2527	474	2.3%	4.2%	13.0%
30510	Creatinine in urine	UCR	511	237	255	19	26	709	368	388	47	3.7%	5.1%	6.6%
30660	Direct bilirubin	BILD	260	186	69	5	9	322	218	115	11	1.9%	3.5%	3.4%
30730	Gamma glutamyltransferase	GGT	97	21	70	6	9	137	29	116	8	6.2%	9.3%	5.8%
30740	Glucose	GLU	145	77	63	5	5	186	96	96	6	3.4%	3.4%	3.2%
30750	HbA1c	HBA1C	455	179	256	20	30	530	202	355	27	4.4%	6.6%	5.1%
30760	HDL cholesterol	HDL	3561	1252	2151	158	258	3515	1491	2536	512	4.4%	7.2%	14.6%
30770	IGF-1	IGF1	4712	1692	2968	52	94	4474	1794	3225	545	1.1%	2.0%	12.2%
30780	LDL cholesterol	LDLD	3260	1249	1906	105	186	3440	1561	2351	472	3.2%	5.7%	13.7%
30790	Lipoprotein A	LPA	1337	495	697	145	175	1487	675	1025	213	10.8%	13.1%	14.3%
30500	Microalbumin in urine	URMA	52	23	24	5	5	74	39	43	8	9.6%	9.6%	10.8%
30810	Phosphate	PHOS	2107	1090	1015	2	4	2412	1254	1342	184	0.1%	0.2%	7.6%
30520	Potassium in urine	URK	205	94	105	6	7	300	145	168	13	2.9%	3.4%	4.3%
30830	SHBG	SHBG	1350	427	873	50	80	1557	506	1161	110	3.7%	5.9%	7.1%
30530	Sodium in urine	URNA	1067	496	525	46	62	1329	696	782	149	4.3%	5.8%	11.2%
30850	Testosterone	TES	1483	807	622	54	85	1814	1131	864	181	3.6%	5.7%	10.0%
30840	Total bilirubin	TBIL	1243	592	606	45	68	1464	732	866	134	3.6%	5.5%	9.2%
30860	Total protein	TP	4895	1611	3266	18	29	4690	1780	3447	537	0.4%	0.6%	11.4%
30870	Triglycerides	TRIG	2331	736	1374	221	311	2569	1063	1948	442	9.5%	13.3%	17.2%
30880	Urate	UA	4017	1675	2293	49	101	4019	1947	2636	564	1.2%	2.5%	14.0%
30670	Urea	BUN	610	346	262	2	5	731	378	374	21	0.3%	0.8%	2.9%
30890	Vitamin D	VITD	2025	916	1058	51	87	2327	1203	1367	243	2.5%	4.3%	10.4%

**FIGURE 2:**
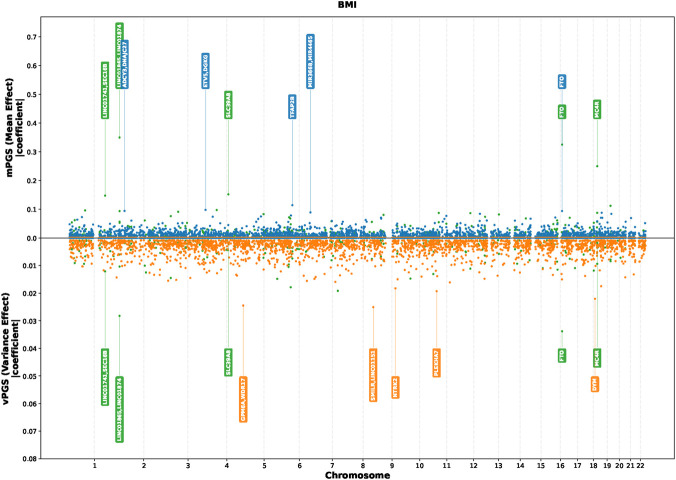
Miami plot showing the model selected mPGS variants (**upper panel**) and vPGS variants (**lower panel**) for BMI plotted by taking the absolute effect size of the variants. The variants that are associated only with the mean BMI are coded as blue dots, variants that are associated only with BMI variability are coded as orange dots and the common variants that are associated with both BMI mean and variability are coded as green dots (both upper and lower panel). Top 5 genes that are mapped to each type of variant with highest effect sizes are annotated and color coded accordingly.

Gene-based annotation results on all traits revealed that a substantial proportion ( > 90% on average) of mPGS and vPGS selected variants were located in the non-coding regions of the human genome, namely: intergenic, intronic, ncRNA and UTR3 (Supplementary Table S3). The overall proportion of variants residing on exonic region ranged from 3% (for GGT: total selected variants = 97, total annotated genes = 137) to 15% (for BILD: total selected variants = 260, total annotated genes = 322) (Table 2; Supplementary Table S3). In the case of BMI for instance, the total number of variants selected by our model were mapped to 4,423 unique genes, and 249 (≈5%) variants were located in the exonic regions (Supplementary Table S3). As expected, the majority of these selected variants reside in intergenic regions (≈46%) , followed by intronic (≈39%) , intronic non-coding RNA (≈6%) , exonic (≈5%) and other non-coding regions (≈4%) of the human genome (Supplementary Table S3). For the variant–gene-based mapping of the overall UK Biobank genotyping dataset used in our analysis, the distribution was as follows: intergenic (≈49%) , intronic (≈36%) , exonic (≈4%) , other (≈11%) . Notably, our models include a variant (rs1421085) at the *FTO* gene locus, which has been found to be strongly associated with fat mass and obesity (Dina et al., 2007; Graff et al., 2017), showing a strong effect on both the mean and the variance of BMI (Figures 1, 2). Different informative variants localized in the same gene (for example, *FTO*) were identified for the mPGS and vPGS models which are likely to be associated with similar biological mechanisms (Figure 2). Likewise, the model reported another top common variant, rs2229616, located at the *MC4R* gene locus—a key regulator of body weight that is common on individuals with penetrant obesity (Riveros-McKay et al., 2019) (Figures 1, 2). Notably, a nonsense mutation at the *MC4R* gene locus introducing a stop codon (p.Tyr35Ter, MAF = 0.01%) has been previously shown to be significantly associated with BMI, where carriers weigh on average ≈7 kg more than non-carriers (Turcot et al., 2018). With further investigation on other blood and urine biomarkers, we could demonstrate that our model was able to identify the most effective genetic loci that are associated with the studied traits (for example: *LPL, LIPG, CETP* for Apolipoprotein A; *LPA, PLG, SLC23* for Lipoprotein A; *PCSK9, LDLR, NECTIN2, LIPG* for cholesterol related biomarkers; *ZPR1, ANGPTL4, MIR122* for triglycerides) (Mack et al., 2017; Cadby et al., 2022; Karjalainen et al., 2024) (Supplementary Material 2). In short, our functional and gene-based annotation results on the model-predicted variants demonstrated that our sparse polygenic models predicted the informative variants residing on the most effective gene loci for all studied biomarkers including BMI (Figure 1 and Supplementary Material 2).

Further, we characterized the linkage between our mPGS and vPGS model selected variants on the basis of LD, the association between alleles of closely linked variants and genes (Pritchard and Przeworski, 2001). In fact, due to linkage disequilibrium, a single genetic variant for a phenotype could be a truly associated variant but also a variant in linkage with the true causal variant. Such relations between variants in the close proximity can lead to multiple statistical (but not causal) associations at nearby variants (Berisa and Pickrell, 2015). The LD patterns across neighboring variants arise due to the fact that the alleles in the nearby loci are inherited together more often than would be expected by chance (Pritchard and Przeworski, 2001). To investigate this, we checked the associations between variants in a locus from the mPGS and the vPGS models to see if there are distinct variants selected by the mean- and the variance-based methods showing a moderate to high correlation $\left(R^{2}\geq 0.5\right)$ between each other (see Methods for details). As expected, we identified multiple genetic variants predicted by two different models that are in linkage $\left(R^{2}\geq 0.5\right)$ sharing a common LD-Block except for three traits: Microalbumin in urine (URMA), Glucose (GLU) and Alkaline phosphatase (ALP). Interestingly, for ALP, there were no shared variants and no variants in linkage between mPGS and vPGS model-selected variants also after taking LD into account (Supplementary Material 2), meaning that our sparse model identified no common genetic variants that are associated with ALP mean and variability. Likewise, for the blood biomarker GLU and the urine biomarker URMA, there were no additional genetic variants in linkage except for those that are commonly identified by the mPGS and vPGS methods. The details of the LD information for all traits is available in Table 2 and Supplementary Material 2.

### 3.4 Gene enrichment of mPGS and vPGS gene-sets

To explore the enrichment of gene sets that are specific to our mPGS and vPGS models, we defined two separate set of genes: i) mPGS gene-set and ii) vPGS gene-set, for all traits and performed gene enrichment analysis. We used GWAS catalog 2025 (Cerezo et al., 2025) to investigate the significance of each specific gene set for all traits (Supplementary Table S2). For BMI, the enrichment analysis revealed a significant enrichment of vPGS gene sets on terms from the GWAS catalog 2025 such as: Adult body size (vPGS: $p=5.2\times 10^{-52},OR=4.4$ ; mPGS: $p=6.3\times 10^{-180},OR=15.37$ ), obesity-related traits (vPGS: $p=1.15\times 10^{-18},OR=2.29$ ; mPGS: $p=2.05\times 10^{-13},OR=2.13$ ), metabolite levels (vPGS: $p=9.09\times 10^{-16},OR=1.73$ ; mPGS: $p=2.27\times 10^{-11},OR=1.65$ ) and BMI- main effects and physical activity interaction (vPGS: $p=1.13\times 10^{-15},OR=6.8$ ; mPGS: $p=2.49\times 10^{-31},OR=16.56$ ) (Supplementary Table S2). Interestingly, we observed that the subsets of gene sets prioritized by vPGS model showed higher significance regarding the response to Amphetamines, a drug known to be associated with obesity (Ricca et al., 2009; Stăcescu et al., 2019), compared to mPGS gene sets (vPGS: $p=9.1\times 10^{-8},OR=7.9$ ; mPGS: $p=6.1\times 10^{-5},OR=5.2$ ), hinting that genes prioritized by vPGS model exhibited significantly stronger enrichment against the response to drug usage. Similar results were noticed also for other traits such as LDL Cholesterol, where vPGS selected gene subsets exhibited relatively stronger enrichment towards the response to Statin therapy $\left(p=6.9\times 10^{-6},OR=3.9\right)$ than those that are selected by mPGS model $\left(p=1.8\times 10^{-3},OR=3.0\right)$ (Supplementary Table S2). These findings suggest that the genetic variants predicted by our vPGS model, that are associated with the variability of a trait, might represent potential genetic components modulating phenotypic sensitivity to environmental perturbations.

## 4 Discussion

Classical PGS models focus mainly on the aggregated effect of genetic variants on the mean of the phenotypic trait of interest. However, variants may also be associated with the variance of a complex phenotypic trait, or other metrics of the phenotype’s distribution. In this study, we simultaneously derived both a mean-based mPGS and a variance-based vPGS model (Equation 1) for BMI, blood and urine biomarkers, traits with a potentially relevant gene–environment component. The addition of the variance component to the PGS framework introduces novel biological insights and potentially identifies individuals who may benefit from targeted environmental interventions, such as lifestyle modification or response to medication. Variants associated with only the mean of the phenotype are genetic susceptibility loci that have reduced sensitivity to the trait variability and variants associated with only the variance of phenotype are loci that are presumed to capture potential gene-environment interaction. In fact, they might be a proxy for strong interaction effect underlying with known risk disease factors, such as sedentary behavior or diet (Wang et al., 2019). Our analyses revealed genetic loci that influence the average BMI and the average blood and urine biomarkers alongside variants that predominantly affect the variability of these studied traits. Notably, some variants contributed to both components, while others were specific to variance, suggesting distinct mechanisms and potential evidence for gene–environment interplay. These findings demonstrate that integrating variance effects into polygenic modeling can improve model interpretability and yield refined predictive insights for complex traits within precision medicine.

Variant and gene overlap analyses based on the underlying linkage disequilibrium (LD) structure between the model-selected variants revealed a substantial degree of intersection between variants and annotated genes estimated by the mean-based PGS (mPGS) and variance-based PGS (vPGS) models, that are significantly associated with the traits under investigation. This further demonstrates the utility of incorporating LD information while performing gene-based overlap analyses. An additional gene enrichment analysis demonstrated a strong enrichment of the annotated trait-specific genes in biological pathways and functional terms that are related to the respective traits analyzed in this study.

To summarize, we demonstrate that our novel framework can simultaneously predict genetic influences on both the mean and variance components of complex multifactorial traits, highlighting significant shared variants and genes between the mPGS and vPGS models. We also reported that *snpboostlss* selected more variants that are associated with the variability of the phenotype compared to those that are associated with the average of the phenotype. This is an expected behavior and also in line with the recent studies showing vQTLs that capture genetic components associated with the phenotypic variability that are not reflected by conventional mean-based approaches (Wang et al., 2019; Xiang et al., 2025). Furthermore, the vPGS model is anticipated to capture genetic variants influencing trait variability with response to several factors like environment, age, sex and metabolite levels that are independent of the additive mean effects. Similar results were also reported recently where the authors identified 67 vQTL variants without having additive mean effects on the average level of protein (Hillary et al., 2024). Also, the prediction performance of the mPGS models showed significant heterogeneity. By comparing the predictive performance of our mPGS models for all analyzed traits with previous reports where the authors used Nagelkerke’s pseudo- $R^{2}$ as a evaluation metric (Sinnott-Armstrong et al., 2021; Tanigawa et al., 2022), we showed comparable predictive accuracy between our sparser models and BASIL algorithm-based models. Notably, the maximum difference on the predictive performance of our mPGS model was observed for BMI (difference $R^{2}=0.06$ ; mPGS $R^{2}=0.04$ , BASIL $R^{2}=0.11$ ). Here, it is also important to note that different evaluation metrics were used to quantify the predictive performance of the models, as it has been previously reported that the choice of the evaluation metric while computing the predictive performance of the models can lead to considerably different results (Staerk et al., 2024).

In conclusion, we performed an in-depth analysis of genetic components by means of polygenic prediction models across BMI and 30 blood and urine biomarkers from the UK Biobank, and reported sparse PGS models that are able to simultaneously predict the genetic variants that are associated with both the average and the variability of the phenotype. While we demonstrated the statistical significance in predictive performance of the PGS models across multiple traits, clinical relevance of the constructed PGS models is not guaranteed. Moreover, the genetic variants that are associated with the variance of the phenotype may not necessarily capture GxE modulations but also other mechanisms such as gene-gene interaction, interaction between SNPs (epistasis), non-linear genetic effects and genetic effects on higher moments of the phenotypic distribution which can also lead to heteroscedasticity. Thus, the presented results need a thorough investigation as well as detailed functional characterization and therefore, should be interpreted with caution. In future, extension of mean-based and variance-based PGS models within a joint modeling framework, and its application to multiple traits can help identify mean and variance associated genetic components providing valuable insights into complex genetic architecture and the underlying biological mechanisms.

## Data Availability

The data analyzed in this study is subject to the following licenses/restrictions: This research has been conducted using the UK Biobank resource under application number 135122 (http://www.ukbiobank.ac.uk). Requests to access these datasets should be directed to UK Biobank, http://www.ukbiobank.ac.uk. Our software code and analysis is available at https://github.com/boost-PRS.
