# Supplementary material for: Polygenic modeling of genetic effects on both phenotypic mean and variance: distributional regression for BMI, blood and urine biomarkers in the UK Biobank
Source: Front Bioinform. 2026 Jun 11;6:1800403. doi: 10.3389/fbinf.2026.1800403 (PMC13294066; doi:10.3389/fbinf.2026.1800403)
Supplement: Supplementary file 3 [file DataSheet1.pdf]

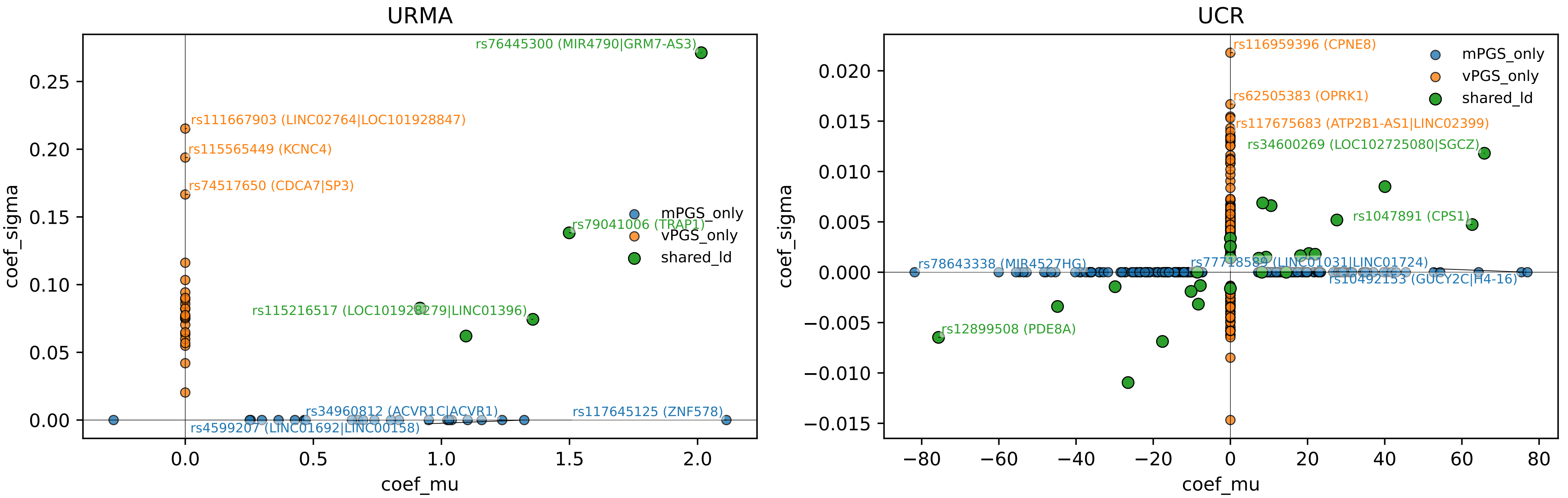

URMA
rs76445300 (MIR4790|GRM7-AS3)
rs111667903 (LINC02764|LOC101928847)
rs115565449 (KCNC4)
rs74517650 (CDCA7|SP3)
rs79041006 (TRAP1)
rs115216517 (LOC101928279|LINC01396)
rs34960812 (ACVR1C|ACVR1)
rs117645125 (ZNF578)
rs4599207 (LINC01692|LINC00158)
mPGS_only
vPGS_only
shared_ld
coef_sigma
coef_mu
0.00
0.05
0.10
0.15
0.20
0.25
0.0
0.5
1.0
1.5
2.0
UCR
rs116959396 (CPNE8)
rs62505383 (OPRK1)
rs117675683 (ATP2B1-AS1|LINC02399)
rs34600269 (LOC102725080|SGCZ)
rs1047891 (CPS1)
rs78643338 (MIR4527HG)
rs77718589 (LINC01031|LINC01724)
rs10492153 (GUCY2C|H4-16)
rs12899508 (PDE8A)
mPGS_only
vPGS_only
shared_ld
coef_sigma
coef_mu
0.020
0.015
0.010
0.005
0.000
−0.005
−0.010
−0.015
−80
−60
−40
−20
0
20
40
60
80

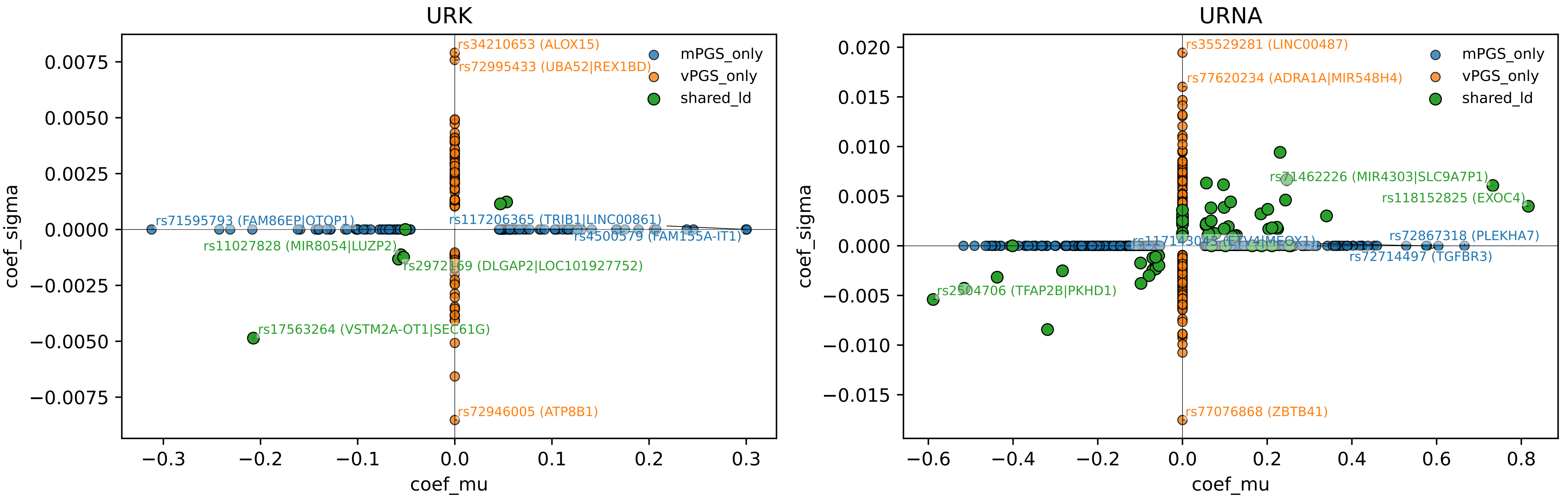
URK
URNA
coef_mu
coef_sigma
mPGS_only
vPGS_only
shared_ld
rs34210653 (ALOX15)
rs72995433 (UBA52|REX1BD)
rs71595793 (FAM86EP|OTOP1)
rs117206365 (TRIB1|LINC00861)
rs4500579 (FAM155A-IT1)
rs11027828 (MIR8054|LUZP2)
rs17563264 (VSTM2A-OT1|SEC61G)
rs72946005 (ATP8B1)
rs35529281 (LINC00487)
rs77620234 (ADRA1A|MIR548H4)
rs71462226 (MIR4303|SLC9A7P1)
rs118152825 (EXOC4)
rs72867318 (PLEKHA7)
rs72714497 (TGFBR3)
rs77076868 (ZBTB41)

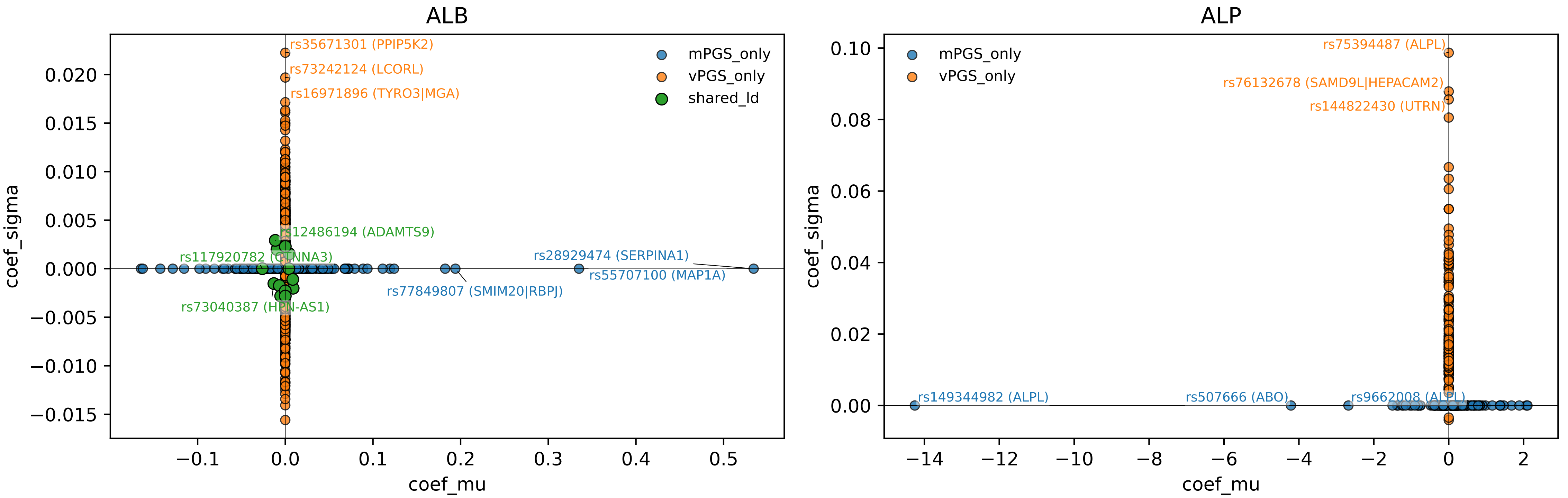

ALB
rs35671301 (PPIP5K2)
rs73242124 (LCORL)
rs16971896 (TYRO3|MGA)
rs12486194 (ADAMTS9)
rs117920782 (CHNNA3)
rs73040387 (HPN-AS1)
rs28929474 (SERPINA1)
rs55707100 (MAP1A)
rs77849807 (SMIM20|RBPJ)
mPGS_only
vPGS_only
shared_ld
coef_sigma
coef_mu
ALP
rs75394487 (ALPL)
rs76132678 (SAMD9L|HEPACAM2)
rs144822430 (UTRN)
rs149344982 (ALPL)
rs507666 (ABO)
rs9662008 (ALPL)
mPGS_only
vPGS_only
coef_sigma
coef_mu

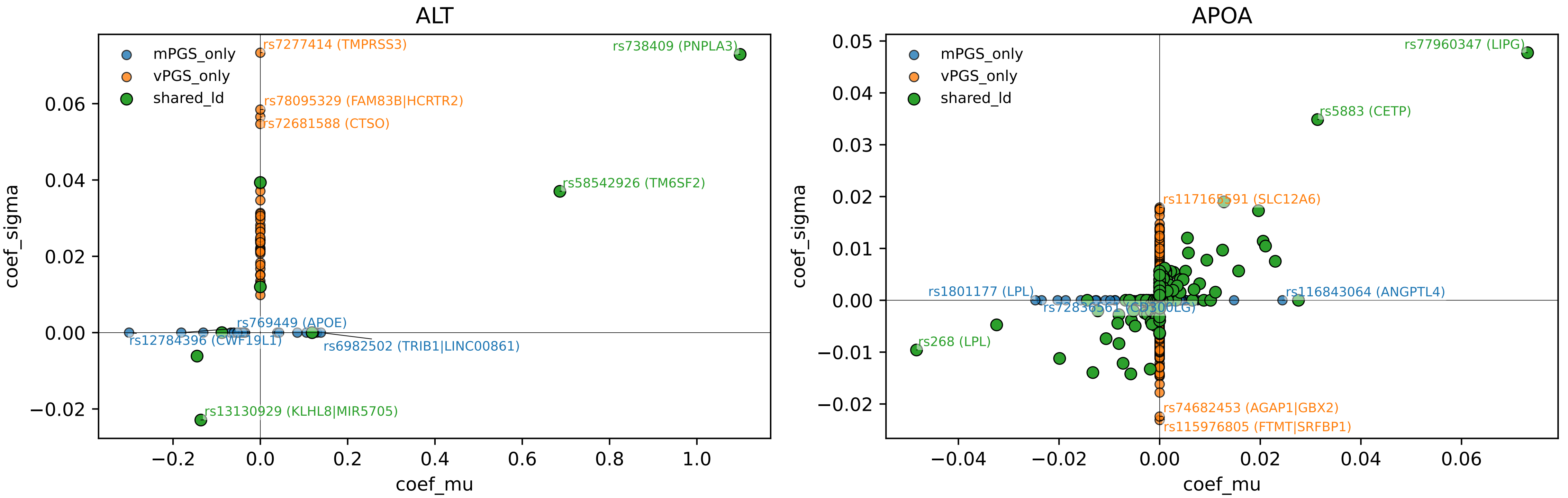

ALT
mPGS_only
vPGS_only
shared_ld
rs7277414 (TMPRSS3)
rs738409 (PNPLA3)
rs78095329 (FAM83B|HCRTR2)
rs72681588 (CTSO)
rs58542926 (TM6SF2)
rs769449 (APOE)
rs12784396 (CWF19L1)
rs6982502 (TRIB1|LINC00861)
rs13130929 (KLHL8|MIR5705)
coef_sigma
coef_mu
APOA
rs77960347 (LIPG)
rs5883 (CETP)
rs117165591 (SLC12A6)
rs1801177 (LPL)
rs116843064 (ANGPTL4)
rs72836561 (CD300LG)
rs268 (LPL)
rs74682453 (AGAP1|GBX2)
rs115976805 (FTMT|SRFBP1)

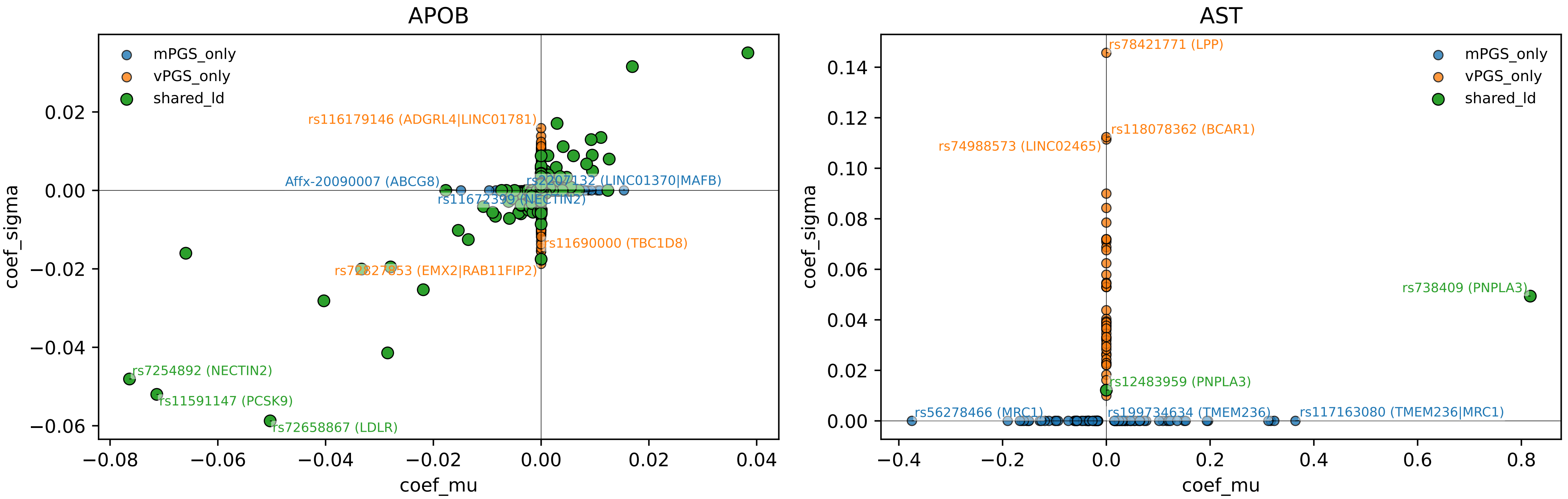
APOB
mPGS_only
vPGS_only
shared_ld
rs116179146 (ADGRL4|LINC01781)
Affx-20090007 (ABCG8)
rs2207132 (LINC01370|MAFB)
rs11672399 (NECTIN2)
rs11690000 (TBC1D8)
rs7254892 (NECTIN2)
rs11591147 (PCSK9)
rs72658867 (LDLR)
coef_sigma
coef_mu
0.02
0.00
−0.02
−0.04
−0.06
−0.08
0.04
AST
rs78421771 (LPP)
rs118078362 (BCAR1)
rs74988573 (LINC02465)
rs738409 (PNPLA3)
rs12483959 (PNPLA3)
rs56278466 (MRC1)
rs199734634 (TMEM236)
rs117163080 (TMEM236|MRC1)
0.14
0.12
0.10
0.08
0.06
0.04
−0.4
−0.2
0.0
0.2
0.4
0.6
0.8

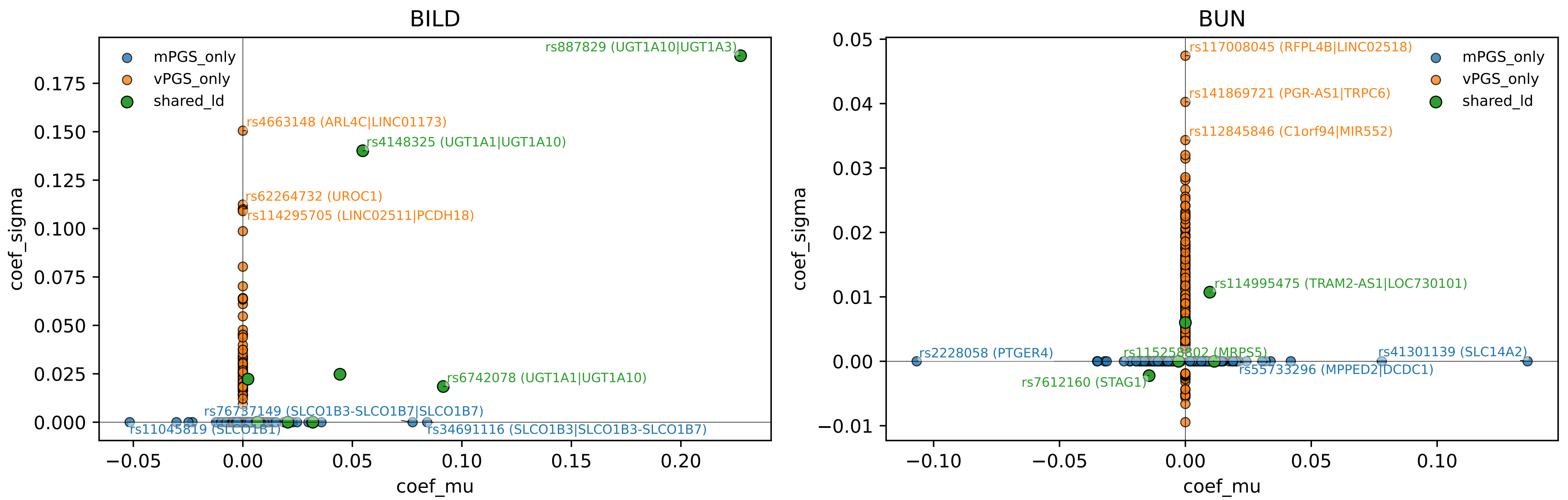
BILD
mPGS_only
vPGS_only
shared_ld
rs887829 (UGT1A10|UGT1A3)
rs4663148 (ARL4C|LINC01173)
rs4148325 (UGT1A1|UGT1A10)
rs62264732 (UROC1)
rs114295705 (LINC02511|PCDH18)
rs6742078 (UGT1A1|UGT1A10)
rs76737149 (SLCO1B3-SLCO1B7|SLCO1B7)
rs11045819 (SLCO1B1)
rs34691116 (SLCO1B3|SLCO1B3-SLCO1B7)
coef_sigma
coef_mu
0.000
0.025
0.050
0.075
0.100
0.125
0.150
0.175
−0.05
0.00
0.05
0.10
0.15
0.20
BUN
rs117008045 (RFPL4B|LINC02518)
rs141869721 (PGR-AS1|TRPC6)
rs112845846 (C1orf94|MIR552)
rs114995475 (TRAM2-AS1|LOC730101)
rs2228058 (PTGER4)
rs115258802 (MRPS5)
rs41301139 (SLC14A2)
rs55733296 (MPPED2|DCDC1)
rs7612160 (STAG1)
coef_sigma
coef_mu
−0.01
0.00
0.01
0.02
0.03
0.04
0.05
−0.10
−0.05
0.00
0.05
0.10

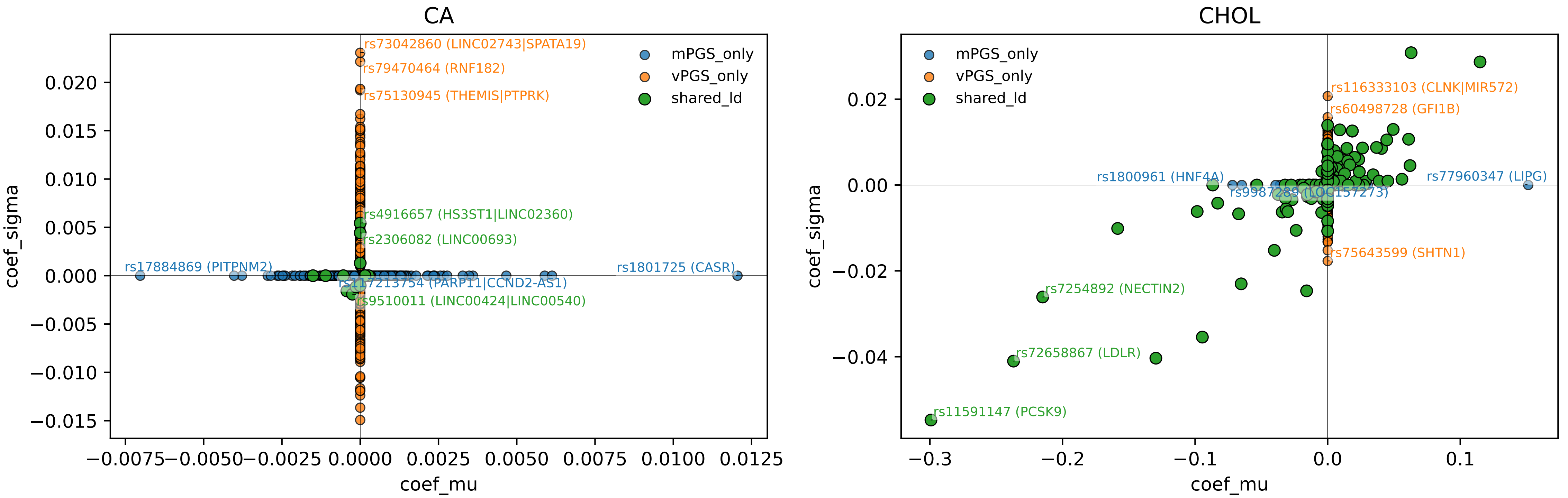

CA
CHOL
mPGS_only
vPGS_only
shared_ld
coef_mu
coef_sigma
rs73042860 (LINC02743|SPATA19)
rs79470464 (RNF182)
rs75130945 (THEMIS|PTPRK)
rs4916657 (HS3ST1|LINC02360)
rs2306082 (LINC00693)
rs17884869 (PITPNM2)
rs1801725 (CASR)
rs117213754 (PARP11|CCND2-AS1)
rs9510011 (LINC00424|LINC00540)
rs116333103 (CLNK|MIR572)
rs60498728 (GFI1B)
rs1800961 (HNF4A)
rs77960347 (LIPG)
rs9987289 (LOC157273)
rs75643599 (SHTN1)
rs7254892 (NECTIN2)
rs72658867 (LDLR)
rs11591147 (PCSK9)

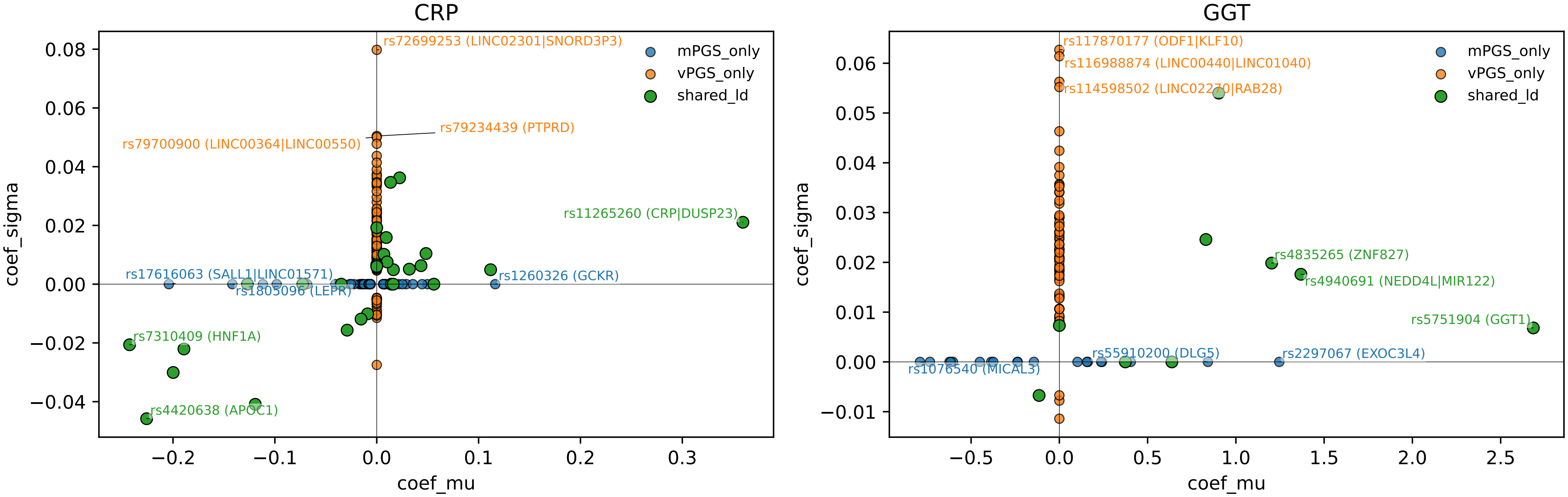

CRP
rs72699253 (LINC02301|SNORD3P3)
rs79234439 (PTPRD)
rs79700900 (LINC00364|LINC00550)
rs11265260 (CRP|DUSP23)
rs17616063 (SALL1|LINC01571)
rs1805096 (LEPR)
rs1260326 (GCKR)
rs7310409 (HNF1A)
rs4420638 (APOC1)
mPGS_only
vPGS_only
shared_ld
coef_sigma
coef_mu
GGT
rs117870177 (ODF1|KLF10)
rs116988874 (LINC00440|LINC01040)
rs114598502 (LINC02270|RAB28)
rs4835265 (ZNF827)
rs4940691 (NEDD4L|MIR122)
rs5751904 (GGT1)
rs55910200 (DLG5)
rs2297067 (EXOC3L4)
rs1076540 (MICAL3)

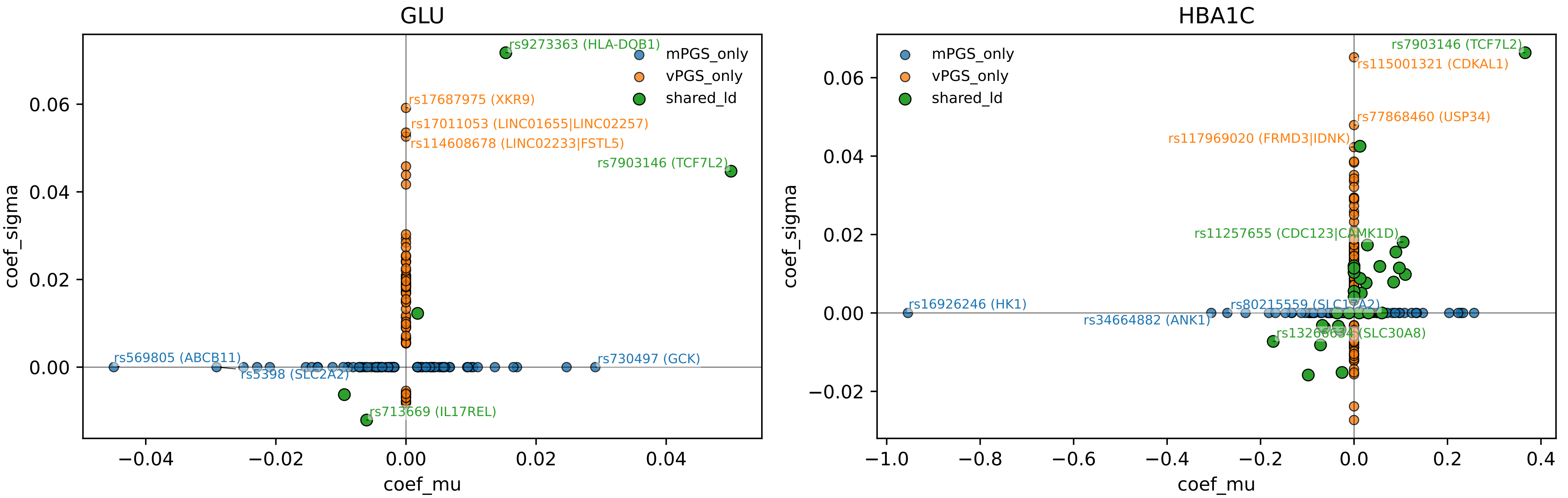

GLU
rs9273363 (HLA-DQB1)
mPGS_only
vPGS_only
shared_ld
rs17687975 (XKR9)
rs17011053 (LINC01655|LINC02257)
rs114608678 (LINC02233|FSTL5)
rs7903146 (TCF7L2)
rs569805 (ABCB11)
rs5398 (SLC2A2)
rs730497 (GCK)
rs713669 (IL17REL)
0.06
0.04
0.02
0.00
−0.04
−0.02
0.00
0.02
0.04
coef_sigma
coef_mu
HBA1C
mPGS_only
vPGS_only
shared_ld
rs7903146 (TCF7L2)
rs115001321 (CDKAL1)
rs77868460 (USP34)
rs117969020 (FRMD3|IDNK)
rs11257655 (CDC123|CAMK1D)
rs16926246 (HK1)
rs80215559 (SLC17A2)
rs34664882 (ANK1)
rs13266634 (SLC30A8)
0.06
0.04
0.02
0.00
−0.02
−1.0
−0.8
−0.6
−0.4
−0.2
0.0
0.2
0.4
coef_sigma
coef_mu

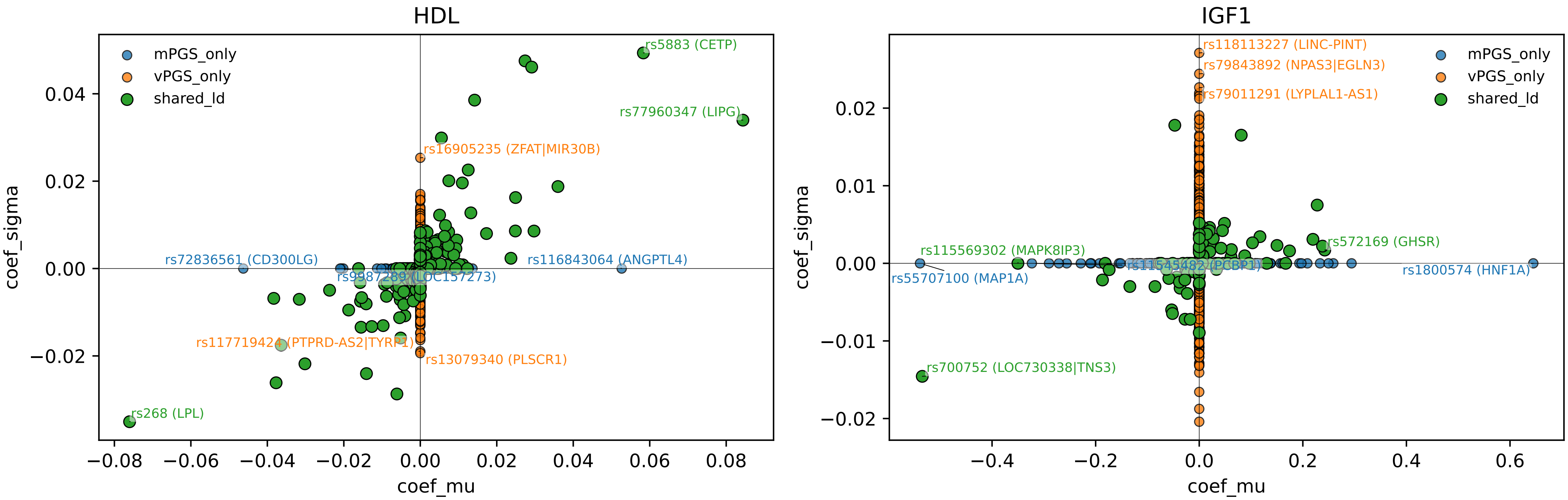

HDL
mPGS_only
vPGS_only
shared_ld
rs5883 (CETP)
rs77960347 (LIPG)
rs16905235 (ZFAT|MIR30B)
rs72836561 (CD300LG)
rs116843064 (ANGPTL4)
rs9987289 (LOC157273)
rs117719424 (PTPRD-AS2|TYRP1)
rs13079340 (PLSCR1)
rs268 (LPL)
coef_sigma
coef_mu
IGF1
rs118113227 (LINC-PINT)
rs79843892 (NPAS3|EGLN3)
rs79011291 (LYPLAL1-AS1)
rs115569302 (MAPK8IP3)
rs572169 (GHSR)
rs55707100 (MAP1A)
rs115545482 (PCBP1)
rs1800574 (HNF1A)
rs700752 (LOC730338|TNS3)

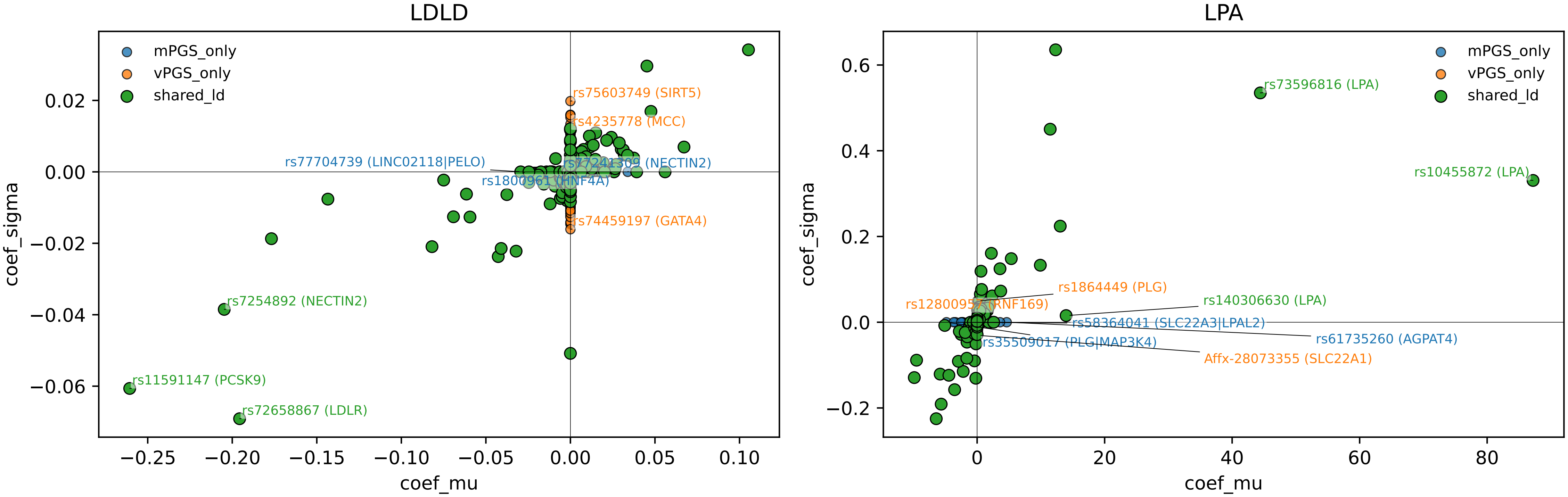

LDLD
mPGS_only
vPGS_only
shared_ld
rs75603749 (SIRT5)
rs4235778 (MCC)
rs77704739 (LINC02118|PELO)
rs1800961 (HNF4A)
rs74459197 (GATA4)
rs7254892 (NECTIN2)
rs11591147 (PCSK9)
rs72658867 (LDLR)
coef_sigma
coef_mu
LPA
mPGS_only
vPGS_only
shared_ld
rs73596816 (LPA)
rs10455872 (LPA)
rs1864449 (PLG)
rs140306630 (LPA)
rs58364041 (SLC22A3|LPAL2)
rs61735260 (AGPAT4)
rs35509017 (PLG|MAP3K4)
Affx-28073355 (SLC22A1)
coef_sigma
coef_mu

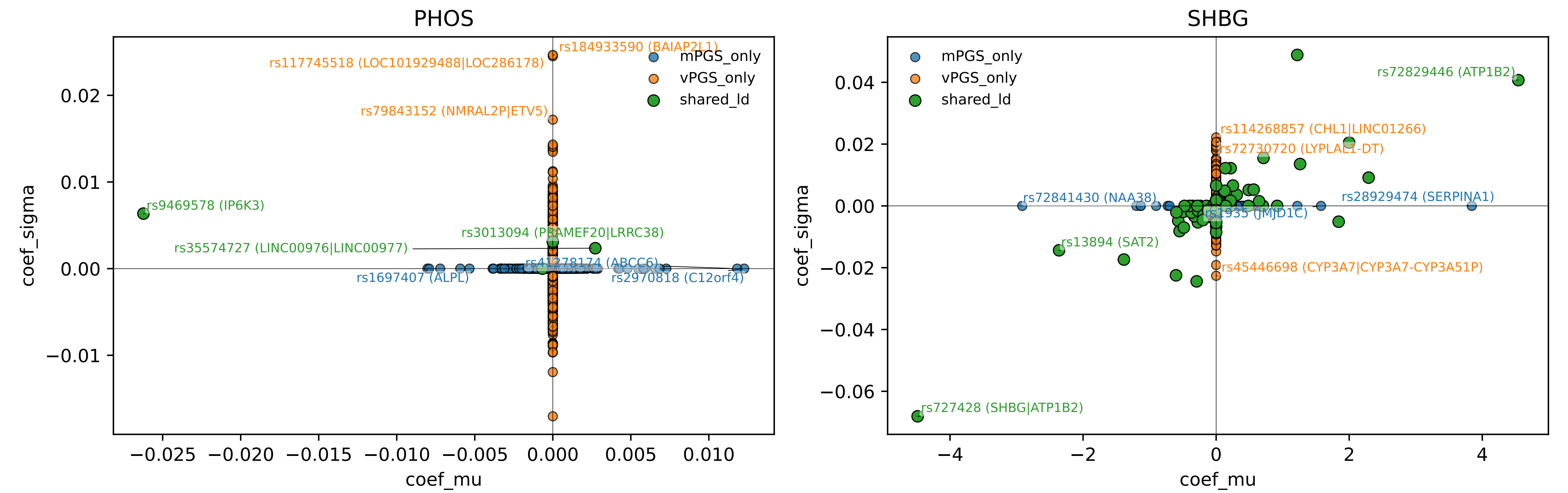

PHOS
SHBG
coef_sigma
coef_mu
mPGS_only
vPGS_only
shared_ld
rs184933590 (BAIAP2L1)
rs117745518 (LOC101929488|LOC286178)
rs79843152 (NMRAL2P|ETV5)
rs9469578 (IP6K3)
rs3013094 (PRAMEF20|LRRC38)
rs35574727 (LINC00976|LINC00977)
rs41278174 (ABCC6)
rs1697407 (ALPL)
rs2970818 (C12orf4)
rs72829446 (ATP1B2)
rs114268857 (CHL1|LINC01266)
rs72730720 (LYPLAL1-DT)
rs72841430 (NAA38)
rs28929474 (SERPINA1)
rs1935 (JMJD1C)
rs13894 (SAT2)
rs45446698 (CYP3A7|CYP3A7-CYP3A51P)
rs727428 (SHBG|ATP1B2)

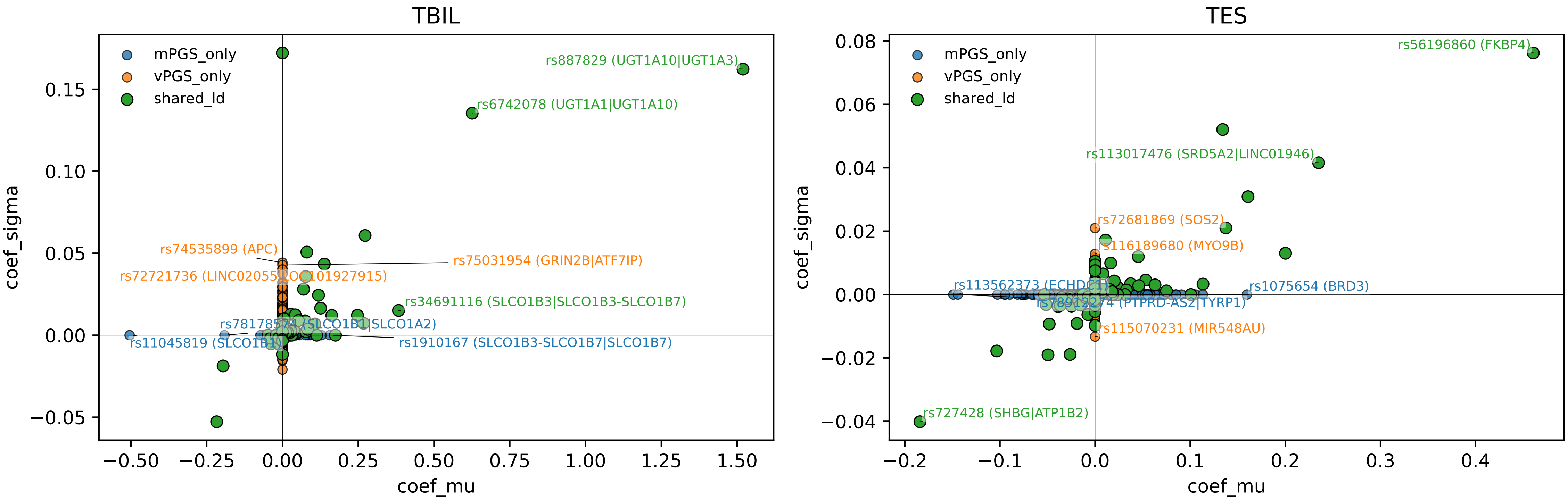
TBIL
mPGS_only
vPGS_only
shared_ld
rs887829 (UGT1A10|UGT1A3)
rs6742078 (UGT1A1|UGT1A10)
rs74535899 (APC)
rs75031954 (GRIN2B|ATF7IP)
rs34691116 (SLCO1B3|SLCO1B3-SLCO1B7)
rs11045819 (SLCO1B1)
rs1910167 (SLCO1B3-SLCO1B7|SLCO1B7)
coef_sigma
coef_mu
0.15
0.10
0.05
0.00
−0.05
−0.50
−0.25
0.00
0.25
0.50
0.75
1.00
1.25
1.50
TES
mPGS_only
vPGS_only
shared_ld
rs56196860 (FKBP4)
rs113017476 (SRD5A2|LINC01946)
rs72681869 (SOS2)
rs116189680 (MYO9B)
rs113562373 (ECHDC1)
rs1075654 (BRD3)
rs115070231 (MIR548AU)
rs727428 (SHBG|ATP1B2)
coef_sigma
coef_mu
0.08
0.06
0.04
0.02
0.00
−0.02
−0.04
−0.2
−0.1
0.0
0.1
0.2
0.3
0.4

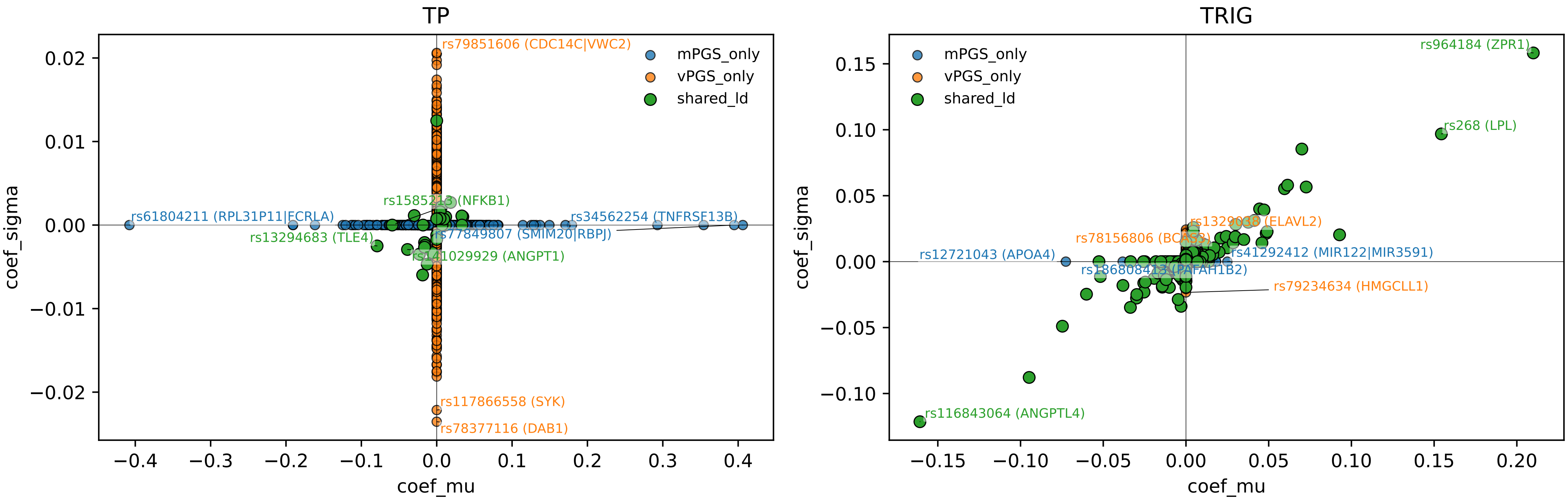
TP
rs79851606 (CDC14C|VWC2)
mPGS_only
vPGS_only
shared_ld
rs1585215 (NFKB1)
rs61804211 (RPL31P11|FCRLA)
rs34562254 (TNFRSF13B)
rs13294683 (TLE4)
rs77849807 (SMIM20|RBPJ)
rs141029929 (ANGPT1)
rs117866558 (SYK)
rs78377116 (DAB1)
coef_sigma
coef_mu
TRIG
rs964184 (ZPR1)
rs268 (LPL)
rs1329038 (ELAVL2)
rs78156806 (BCAS3)
rs12721043 (APOA4)
rs41292412 (MIR122|MIR3591)
rs186808413 (PAFAH1B2)
rs79234634 (HMGCLL1)
rs116843064 (ANGPTL4)

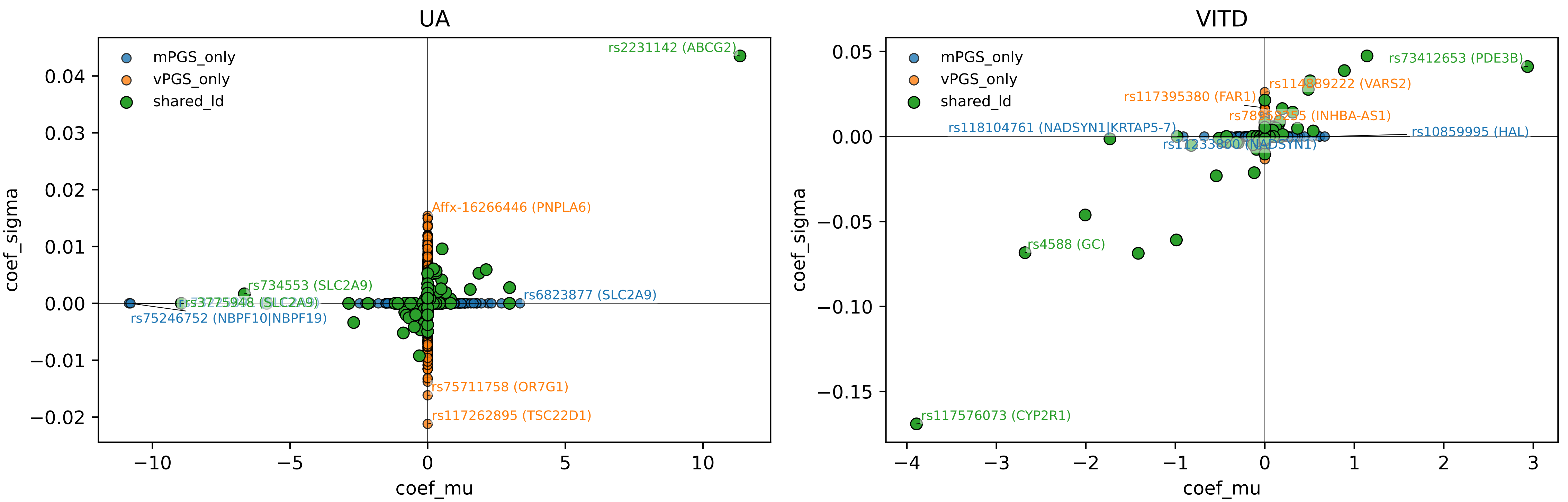
UA
mPGS_only
vPGS_only
shared_ld
rs2231142 (ABCG2)
Affx-16266446 (PNPLA6)
rs734553 (SLC2A9)
rs6823877 (SLC2A9)
rs75246752 (NBPF10|NBPF19)
rs75711758 (OR7G1)
rs117262895 (TSC22D1)
coef_sigma
coef_mu
VITD
rs73412653 (PDE3B)
rs114889222 (VARS2)
rs117395380 (FAR1)
rs118104761 (NADSYN1|KRTAP5-7)
rs10859995 (HAL)
rs4588 (GC)
rs117576073 (CYP2R1)
